# Biomonitoring of Occupational Exposure to Mercury Among Dental Health Workers in LMICs: A Systematic Review

**DOI:** 10.1016/j.identj.2026.109560

**Published:** 2026-05-20

**Authors:** Imane Bensouda Korachi, Aziza Menouni, Radu Corneliu Duca, Lode Godderis, Samir El Jaâfari, Younes Filali-Zegzouti

**Affiliations:** a Human Epidemiology and Environmental Health Research Team, NRHE laboratory, Moulay Ismail University of Meknes, Meknes, Morocco; b Environment and Health Unit, Department of Public Health and Primary Care, Katholieke Universiteit Leuven (KU Leuven), Leuven, Belgium; c Environmental Hygiene and Human Biological Monitoring Unit, Department of Health Protection, National Health Laboratory (LNS), Dudelange, Luxembourg; d Department Health Protection, Health Directorate, Strassen, Luxembourg; e IDEWE, External Service for Prevention and Protection at Work, Heverlee, Belgium

**Keywords:** Dentistry, Dental amalgam, Mercury, Biomonitoring, Occupational health, Health effects

## Abstract

Mercury exposure remains a significant occupational hazard for dental health workers, especially in low- and middle-income countries (LMICs), due to the continued use of dental amalgam. This systematic review synthesizes the existing evidence on mercury exposure in dental professionals, focusing on biomonitoring data and associated health outcomes in LMICs. In December 2024, a comprehensive literature search was conducted across three electronic databases, PubMed, Scopus and Web of Science to identify primary research studies published from January 2014 onwards. Studies assessing occupational mercury exposure through biomonitoring in dental health workers were included. Nine studies, encompassing 524 dental professionals, met the inclusion criteria. Mercury biomonitoring was conducted using blood (n = 3), urine (n = 7) and hair samples (n = 2). Most studies reported elevated mercury levels in dental workers compared to control groups. Environmental monitoring data further indicated that mercury vapor concentrations in dental clinics may exceed recommended safety thresholds. However, current evidence regarding potential health effects associated with occupational exposure to mercury is inconclusive due to methodological limitations. Mercury remains an occupational hazard for dental health workers in LMICs. As dental amalgam continues to be used in resource-limited settings, implementing biomonitoring programs could enable early detection of mercury-related health issues, protecting workers’ health until global mercury phase-down goals are achieved.

## Introduction

Mercury exposure remains a global health concern.^1^ In response, the Minamata Convention was adopted in 2013 to protect human health and the environment from anthropogenic emissions and releases of mercury and mercury compounds.^2^ Among those sources, dental applications contribute an estimated 226 to 322 tons annually, equivalent to about 6% of total global mercury consumption.^3^ Accordingly, Article 4 of the Minamata Convention supports the implementation of the phase-down of dental amalgam, an effort aimed at reducing mercury use in dentistry due to environmental and health concerns.^2^

Dental amalgam, a material that has been in use for over 175 years, remains a staple in restorative dentistry.^4^ It is particularly valued for its affordability, durability and reliability, especially under challenging clinical conditions.^4^^,^^5^ As a result, it continues to be widely used, especially in low- and middle-income countries (LMICs), driven by factors such as high caries prevalence, cost-effectiveness and the availability of alternatives.^6^ However, concerns about its safety remain, with the most frequently cited issues being environmental contamination and potential adverse health effects resulting from the release and systemic absorption of mercury.^7^, ^8^, ^9^ In the case of dental professionals, the use of dental amalgam poses a risk of occupational mercury exposure, as they are regularly exposed to mercury during the mixing process in the amalgamator, as well as during the insertion, condensation and removal of amalgam restorations.^10^, ^11^, ^12^

Implementing the phase-down of dental amalgam is not without challenges. In its 2021 report on monitoring global progress in reducing the use of dental amalgam, the WHO Oral Health Program found that 84% of the 80 countries surveyed were still using dental amalgam.^13^ Amalgam fillings made up most of the dental restorative materials used in the public sector in up to 36% of LMICs versus only 9% in high-income countries. 40% and 23% of low-income and lower-middle-income countries respectively had no plan to phase down amalgam use. Reported challenges and barriers to phasing down dental amalgam use include dentists’ preference for amalgam due to its ease of use, durability and familiarity; limited funding or low priority for phase-down plans in national health policies; and patients’ preference driven by lower cost, easier access and better insurance coverage compared to mercury-free alternatives.^13^

Recognizing that the Minamata Convention presented a milestone in global efforts to reduce exposure to mercury and the continuous evolution of dental practices, this systematic review aims to provide an up-to-date synthesis of the current evidence base on the occupational exposure of dental workers to mercury, focusing on biomonitoring levels considering current dental practices and dental amalgam use recommendations. Specifically, we will (1) report on the range of concentrations of mercury in biological matrices of dental health professionals, (2) identify factors influencing biomonitoring levels and (3) identify potential health outcomes associated with the use of mercury in dental practices.

## Methods

The following systematic review was reported according to the Preferred Reporting Items for Systematic Reviews and Meta-Analyses (PRISMA) guidelines. The protocol was registered in the International Prospective Register of Systematic Reviews (PROSPERO), registration number CRD42024599017.

### Search strategy

We searched three electronic databases (PubMed, Scopus and Web of Science) for primary research published from January 2014 onwards. This timeframe is informed by the signing of the Minamata Convention in 2013, which advocates for the phase-down of dental amalgam use.^2^ This approach aims to assess current exposure levels within the context of evolving regulatory practices and guidelines concerning mercury use in dentistry. Search terms were developed using keywords for “dental health workers” and “mercury” separated by Boolean operators (Supplementary Material 1). Reference lists of relevant original publications and review articles retrieved through the search strategy were scanned for additional articles. The search was also rerun before the final analysis, but no additional papers were retrieved for inclusion.

### Study eligibility and selection criteria

Articles were uploaded to the Covidence systematic review management tool (www.covidence.org), and duplicates were removed automatically. Two reviewers (IBK & AM) independently screened titles and abstracts and performed full-text screening using Covidence based on inclusion/exclusion criteria. Inclusion and exclusion criteria were defined using the Population, Exposure, Comparator, Outcome and Study design (PECOS) framework: (P) dental health workers; (E) exposure to mercury through the preparation, manipulation or waste disposal of dental amalgam; (C) nondental healthcare professionals, (O) mercury levels of dental health workers assessed through biomarkers of exposure in human matrices (mercury in hair, urine and blood) and (S) observational studies (cross-sectional, case-control, cohort). Publications reporting nonoccupational exposure, in vitro or animal studies, or out of topic were excluded from further analysis.

### Data extraction

For each included study, one reviewer (IBK) independently extracted the data using an Excel form with predefined data points. A second reviewer (AM) checked the data extraction for accuracy. Extracted data included study characteristics (author, publication date, country of study, study design, sample size, sampling period), population characteristics for both exposed and control groups (demographics, work environment), mercury exposure levels (biomonitoring data including the matrices, biomarkers of exposure and levels reported, analytical methods, environmental monitoring) and health outcomes (biomarkers of effect, self-reported health effects, correlation with exposure levels) if available.

### Quality assessment

We assessed the quality of studies using the Office of Health Assessment and Translation tool,^14^ evaluating the following domains: selection bias, confounding bias, detection bias, attrition bias, selective reporting bias and statistical bias. For each study, we assigned a risk of bias rating for each domain as either definitely low risk, probably low risk, probably high risk or definitely high risk. For selection bias, studies were rated as definitely low risk if they clearly described a similar recruitment process for both exposed and nonexposed groups (eg, recruited within the same hospital or based on the same inclusion and exclusion criteria). For confounding bias, studies must report nonoccupational exposure sources to mercury (ie, fish intake^15^ and tooth restoration with amalgam fillings^16^) to be rated as definitely low risk. For detection bias, studies that employed a reliable instrument for mercury analysis and reported on the instrument's performance parameters were considered definitely low risk. Studies that reported all measured outcomes (both primary and secondary) as outlined in the protocol, methods, abstract or introduction, were rated as definitely low risk for selective reporting bias. Two reviewers (IBK & AM) carried out quality assessments. Disagreements at any stage were resolved through consensus among the reviewers.

### Data synthesis

A meta-analysis was initially planned but was ruled out due to the small number of studies meeting the inclusion criteria and considerable methodological heterogeneity, including differences in selected biomarkers, units of mercury measurement, assessed health outcomes and the limited quality of the included studies as determined through the quality assessment. The findings are therefore presented as a narrative synthesis.

## Results

### Overview of included studies

**Fig. 1:**
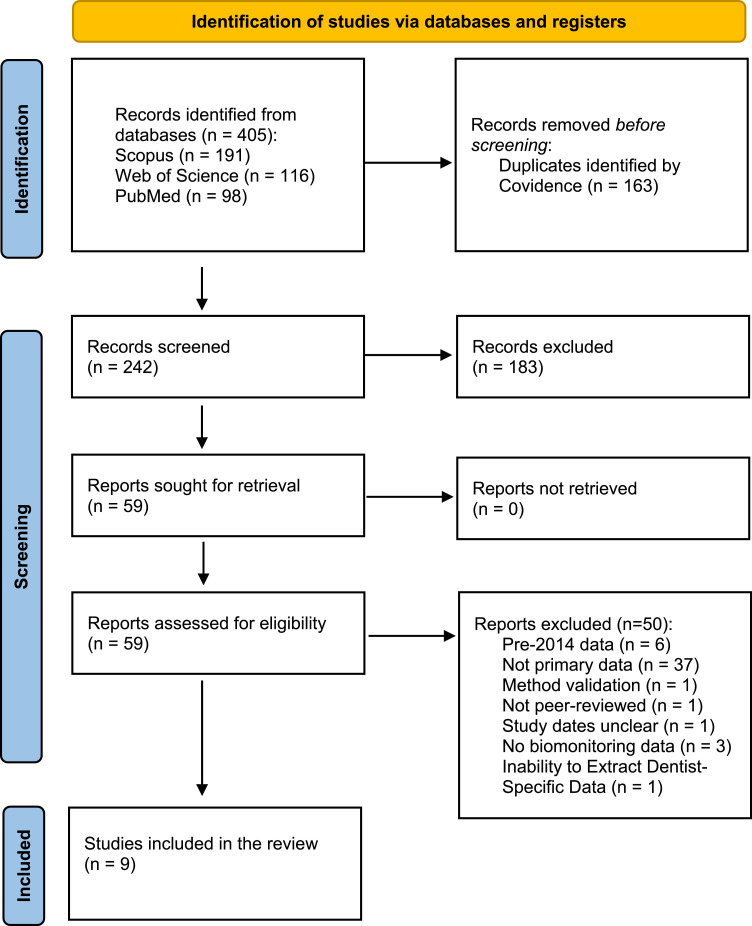
PRISMA flowchart.

A total of 405 records were retrieved through a database search, from which nine studies involving 524 dental professionals and 379 controls were included in the final analysis^17^, ^18^, ^19^, ^20^, ^21^, ^22^, ^23^, ^24^, ^25^ (Figure 1—PRISMA Flowchart). Most studies were performed in Asia (n = 6),^17^^,^^19^, ^20^, ^21^^,^^23^^,^^24^ while two were carried out in African countries,^18^^,^^25^ and only one in Europe^22^ (Table 1). Most of the papers retrieved for this review (n = 7) reported the use of urine as a biological matrix to assess occupational exposure to mercury,^17^, ^18^, ^19^^,^^21^^,^^22^^,^^24^^,^^25^ while blood (n = 3)^17^^,^^20^^,^^24^ and hair (n = 2)^22^^,^^23^ were investigated to a lesser extent. Four studies assessed health outcomes using biomarkers of effects.^17^, ^18^, ^19^^,^^24^ These biomarkers were used to evaluate oxidative stress (n = 3),^17^^,^^18^^,^^24^ renal function (n = 2),^17^^,^^24^ liver function (n = 1)^17^ and immune response (n = 1).^19^ Other health outcomes studied included pregnancy outcomes (n = 1)^18^ and self-perceived health outcomes (n = 1).^21^

**Table 1:** Summary of included studies.

Reference	Study design	Sample size	Exposure measurement						Health outcomes	Main conclusions
			Exposure biomarker*/matrix	Analytical method	Results†			Environmental monitoring		
					Exposed	Controls	Unit			
Jamil et al.^20^*Pakistan*	Cross-sectional	98 37 dentists 31 dental assistants 30 controls	B-Hg/ Plasma	ICP-OES	• Dentists: 29.83 (20.10) • Dental assistants: 22.80 (10.71)	3.28 (1.46)	μg/L	Mean (SD) *(mg/L)* • Samples collected from the discharge point into the wastewater collection system: 173.3 (56.54)x• Samples collected from the side-holding tank of dental chairs: 243.9 (58.23)	Not reported	• Elevated blood mercury in older and more experienced dentists and dental assistants indicates chronic occupational exposure to elemental mercury vapours. • Total mercury concentrations in dental wastewater samples exceed the recommended discharge limit.
Al-Zubaidi et al.^17^*Iraq*	Cross-sectional	35 30 dentists and dental assistants 5 controls	U-Hg/ Urine	CVAAS	4.30 (2.79)	0.57 (0.58)	μg/L	Mean (Se) *(μg/m^3^)* • Mercury vapor in ambient air of 4 dental clinics measured seasonally: from 84.7 (18.67) to 609.3 (238.90) • The highest values found above the work surface and around the patient chair.	• Increased levels of serum cholesterol, plasma aspartate aminotransferase, and alanine aminotransferase in the exposed group. • Significant decrease in plasma concentration of total protein and glutathione levels in dental workers.	• Exposure of dental workers to mercury vapor can induce free radicals, and significant hazardous alterations in some enzymatic activities, liver functions, renal functions.
			B-Hg/ Serum	Direct Mercury Analyzer	1.24 (0.71)	0.19 (0.27)	μg/L			
El-Badry et al.^18^*Egypt*	Cohort	124 64 pregnant dental staff 60 pregnant controls	U-Hg/ Urine	CVAAS	42.27 (14.48)	6.53 (3.80)	μg/g Cr	Highest mean environmental mercury recorded in each trimester (SD) *(μg/m³)*: ◦ 1^st^ trimester: 18.0 (0.9) ◦ 2^nd^ trimester: 19.0 (0.2) ◦ 3^rd^ trimester: 17.0 (0.6)	• Pregnant dental staff had a significantly higher mean urinary mercury level measured in the three trimesters and lower blood antioxidant enzyme activity compared to the nonexposed group. • Pregnant dental staff carried a higher risk of spontaneous abortion (RR 3.52, 95% CI 1.29 to 2.23), and preeclampsia (RR 3.67, 95% CI 1.25 to 10.76), and gave birth to smaller-for-gestational-age babies (RR 6.2, 95% CI 2.3 to 16.4).	• Pregnant dental staff carried a higher risk of adverse pregnancy outcomes, which may be linked to oxidative stress induced by mercury exposure.
Wijesekara et al.^23^*Sri Lanka*	Cross-sectional	100 50 dentists 50 controls	H-Hg/ Hair	FS-AAS	5.36 (2.64)	3.1 (1.99)	ng/mL	Not performed	Not reported	• No occupational hazards associated with using mercury in dental amalgam fillings among dentists working in the government sector in Sri Lanka.
Yazdanian et al.^24^*Iran*	Cross-sectional	57 30 dentists and dental assistants 27 controls	B-Hg/ Total blood	HG-AAS	6.03 (2.22)	4.23 (2.04)	μg/L	Not performed	• Significant reduction in blood superoxide dismutase levels in exposed dental workers. Higher urine creatine levels in exposed.	• Using dental amalgams can lead to the accumulation of Hg in blood and urine and may pose perturbation in oxidative stress index.
			U-Hg/ Urine	HG-AAS	5.7 (3.32)	3.60 (2.16)	μg/L			
Tuček et al.^22^*Czech Republic*	Cross-sectional	50 40 dentists 10 controls	U-Hg/ Urine	AAS	Mean (min.-max.) 3.05 (<LOD-17.14)	Mean (min.-max.) 0.55 (< LOD-2.74)	μg/g Cr	Not performed	Not reported	• Current dental exposures do not exceed acceptable risk levels for dental personnel and are below the biological limit of mercury in urine valid for occupationally exposed persons.
			H-Hg/ Hair	AAS	0.38 (0.060-1.628)	0.23 (0.059-0.453)	μg/g			
Girgin et al.^19^*Turkey*	Cross-sectional	300 135 dental technicians 165 controls	U-Hg/ Urine	ICP-MS	3.60 (1.74)	Not reported	μg/L	Not performed	• Positive correlation between U-Hg and serum neopterin levels of dental technicians. • Lower neopterin, kynurenine and kynurenine to tryptophan ratio in dental technicians.	• Lower neopterin and Kyn/Trp levels in dental technicians may indicate a possible immune-suppressive effect of low-level occupational Hg exposure below safety limits.
Zebbiche et al.^25^*Algeria*	Cross-sectional	42 28 dentists 14 dental assistants	U-Hg/ Urine	ICP-MS	Geometric mean (min, max) 0.82 (<LOQ, 14.05)	NA	μg/g Cr	Not performed	Not reported	• Reduction in occupational mercury exposure is due to a shift from dental amalgam in favour of modern dental materials.
Mawari et al.^21^*India*	Cross-sectional	93 65 dental workers 32 controls	U-Hg/ Urine	ICP-MS	0.51 (1.4)	0.29 (0.28)	μg/L	• Air sampling: 3.56 (3.88) μg/m^3^ • Time range: 227.18 (60.78) minutes	• Significant correlation between Hg exposure and confusion, forgetfulness, chronic constipation, fatigue, arthralgia, insomnia, irritability, change in behaviour, food allergy and chest pain.	• While mercury exposure levels were below the TLV, a significant proportion of healthcare workers exposed to mercury vapours reported health symptoms that could be related to mercury exposure.

⁎ B-Hg, mercury in blood; U-Hg, mercury in urine; H-Hg, mercury in hair.

† Results expressed as mean (SD) unless specified otherwise.

### Mercury exposure

#### Mercury levels in blood

Three studies assessed mercury concentrations in blood.^17^^,^^20^^,^^24^ Jamil et al.^20^ reported the highest mean concentrations of mercury in blood, with 29.83 ± 20.10 μg/L in dentists and 22.80 ± 10.71 μg/L in dental assistants. In contrast, the lowest mean was reported by Al-Zubaidi et al., finding 1.24 ± 0.71 μg/L in the blood of dentists.^17^ All three studies found higher means in exposed groups (ie, dental practitioners) compared to controls, with levels being 8.12 times higher in the blood of dental workers in Pakistan,^20^ 6.5 times higher in Iraq,^17^ and 1.43 times higher in Iran.^24^

#### Mercury levels in urine

Of the seven studies assessing urinary concentrations of mercury, three adjusted for creatinine excretion to take urinary dilution into account,^18^^,^^22^^,^^25^ while no adjustment was made in the remaining four studies.^17^^,^^19^^,^^21^ The highest mean mercury in the urine of dental workers was found in El-Badry’s study with a mean of 42.27 ± 14.48 μg/g Cr.^18^ One study^25^ expressed results in geometric mean, therefore limiting any comparison with other studies. Five studies found higher mercury levels in the urine of dental workers compared to controls,^17^^,^^18^^,^^24^ with levels being 6.47 times higher in Egypt,^18^ 7.54 times in Iraq,^17^ 5.54 times in Czech Republic,^22^ 1.58 times in Iran,^24^ and 1.76 times in India,^21^ while one study did not use a control group,^25^ and another one did not report on mercury measurements in controls.^19^

#### Mercury levels in hair

Two studies assessed mercury levels in hair.^22^^,^^23^ In Sri Lanka, dentists had higher mercury in hair concentrations than controls (5.36 ± 2.64 ng/mL vs. 3.1 ± 1.99 ng/mL).^23^ Similarly, Tuček et al.^22^ reported mercury concentrations of 0.38 μg/g (range: 0.060-1.628) in exposed individuals and 0.23 μg/g (range: 0.059-0.453) in controls.

#### Environmental monitoring

Four studies performed environmental monitoring, of which three used air samples,^17^^,^^18^^,^^21^ and one used wastewater samples.^20^

Measurements taken in Iraqi dental clinics showed concentrations of mercury vapor in ambient air varying from 84.7 ± 18.67 μg/m^3^ to 609.3 ± 238.90 μg/m^3^.^17^ The highest values were found above the work surface and around the patient’s chair. No seasonal variation was observed. In Egypt, monitoring of mercury in the air was performed every trimester of pregnancy.^18^ The highest mean environmental mercury levels recorded in each trimester were 18.0 ± 0.9 μg/m³ for the first trimester, 19.0 ± 0.2 μg/m³ for the second, and 17.0 ± 0.6 μg/m³ for the third trimester. In India, air monitoring was performed in dental clinics for 227.18 ± 60.78 minutes on average, with an average mercury concentration of 3.56 ± 3.88 μg/m^3^.^21^ Measurements taken in dental clinics’ treatment rooms were higher than the control sample.

Jamil et al.^20^ assessed the contribution of dental amalgam in environmental contamination with mercury and found all wastewater samples collected in Pakistani dental practices to be over the local discharge limits. The mercury release into wastewater resulted from amalgam residues and waste from filling placement and removal, with no mercury recycling and/or separation activities involved.

### Factors influencing biomonitoring levels

Regarding mercury levels in the blood, Jamil et al.^20^ found a significant association with age, occupation (dentists vs. dental assistants), working experience, number of hours worked per day, number of amalgam fillings placed per week, and number of own amalgam fillings.

Girgin et al*.*^19^ found no association between years of experience and mercury levels in urine. Tuček et al.^22^ found no correlation between the number of amalgam fillings applied and mercury levels in dentists' urine. On the other hand, Zebbiche et al*.*^25^ found that mercury levels in Algerian dentists' urine were significantly associated with chewing gum, fish consumption and floor cleaning methods.

In Sri Lanka, no correlation was found between mercury hair levels and work factors such as the number of years of service in dentistry, the number of amalgam restorations carried out within 1 week or wearing protective equipment.^23^ Similarly, Tuček et al.^22^ found no association between hair mercury levels and occupational exposure to mercury among Czech dentists.

### Health outcomes

#### Biomarkers of effects

Four studies reported on biomarkers of effects (Table 2).^17^, ^18^, ^19^^,^^24^

**Table 2:** Summary of studies reporting on biomarkers of effects.

Study	Biomarker of effect	Biological matrice	Analytical method	[]		
				Exposed	Controls	Unit
Al-Zubaidi et al.^17^*Iraq*	Cholesterol	Serum	Not reported	140.5 (27.16)	114.80 (8.52)	mg/dL
	Aspartate aminotransferase			29.56 (4.60)	23.21 (2.17)	U/L
	Alanine aminotransferase			32.06 (10.62)	22.60 (3.91)	U/L
	Alkaline phosphatase			58.87 (20.32)	70.60 (3.49)	U/L
	Creatinine			27.27 (5.09)	23.20 (2.15)	mg/dL
	Urea			1.031 (0.38)	0.784 (0.07)	mg/dL
	Total protein			6.78 (0.49)	7.32 (0.48)	g/dL
	Reduced glutathione			5.89 (0.93)	7.45 (0.25)	μmol/L
El-Badry et al.^18^*Egypt*	Glutathione peroxidase	Blood	Enzymatic assay	48.1 (6.6)	66.4 (5.4)	U/g Hb
	Superoxide dismutase			688.4 (87.6)	822.6 (66.4)	U/g Hb
Yazdanian et al.^24^*Iran*	Superoxide dismutase	Serum	ELISA	59.4 (8.75)	64.69 (8.56)	IU/L
	N-Acetyl-β-D-glycosaminidase	Urine		4.35 (0.49)	4.24 (0.91)	IU/L
	Microalbumin		Turbidimetry	6.28 (2.82)	76.68 (290.89)	mg/L
	Immunoglobulin G			1.86 (6.23)	0.47 (0.13)	mg/dL
	Creatinine		Colorimetric assay	948.55 (463.09)	711.40 (236.07)	mg/24h
Girgin et al.^19^*Turkey*	Neopterin	Serum	ELISA	6.69 (3.62)	7.48 (2.57)	nmol/L
	Tryptophan		HPLC	66.77 (12.26)	64.98 (10.53)	μmol/L
	Kynurenine			2.19 (0.56)	2.45 (0.59)	μmol/L
	Kyn/Trp ratio	N/A	N/A	32.87 (7.23)	37.93 (9.43)	μmol/mmol
	Alanine aminotransferase	Serum	Not reported	24.8 (13.94)	Not reported	U/L
	Aspartate aminotransferase			23.1 (9.12)	Not reported	U/L

Three studies reported on mercury-induced oxidative stress,^17^^,^^18^^,^^24^ showing reduced antioxidant activity in dental professionals exposed to mercury. El-Badry et al.^18^ reported decreased activity of blood antioxidant enzymes in pregnant dental professionals, while Yazdanian et al.^24^ observed significantly lower superoxide dismutase activity, a key marker of oxidative stress, in dental workers compared to controls. Al Zubaidi et al.^17^ found a decrease in antioxidant glutathione among dental workers.

Two studies assessed renal function.^17^^,^^24^ Yazdanian et al.^24^ found no association between elevated mercury levels in the blood and urine of dentists and markers of renal dysfunction. These findings were consistent with those of Al Zubaidi et al.,^17^ who reported a nonsignificant increase in blood urea and creatinine levels in dental professionals compared to controls.

One study evaluated liver function and found significant changes in dental workers exposed to mercury, reporting increased cholesterol levels (140.5 ± 27.16 mg/dL vs. 114.80 ± 8.52 mg/dL) and elevated transaminase levels, including plasma aspartate aminotransferase (29.56 ± 4.60 U/L vs. 23.21 ± 2.17 U/L) and alanine aminotransferase (32.06 ± 10.62 U/L vs. 22.60 ± 3.91 U/L) in dental workers compared to controls.^17^

One study assessed immune response and found an immune-suppressive effect in dental technicians with low-level occupational mercury exposure. Girgin et al.^19^ observed lower levels of neopterin and kynurenine pathway parameters, suggesting a potential immune-suppressive effect from mercury exposure below safety limits.

#### Other health outcomes

El-Badry et al.^18^ investigated pregnancy outcomes among pregnant dental professionals and reported higher rates of spontaneous abortion (RR: 3.52; *P* < *.001*) and preeclampsia (RR: 3.67; *P* < .001) compared to the control group. Furthermore, babies born to women in the exposed group tended to be smaller for gestational age (RR: 6.2; *P* < .001) than the unexposed group.

Mawari et al*.*^21^ assessed the general health effects of occupational mercury exposure and found statistically significant correlations between the exposure and symptoms such as confusion, forgetfulness, chronic constipation, fatigue, arthralgia, insomnia, irritability, behavioural changes, food allergies and chest pain.

### Quality assessment

The quality assessment of the included studies revealed several methodological limitations (Supplementary Material 2). Regarding selection criteria, one study did not include a control group in its methodological design.^25^ Three studies did not provide information on the selection and/or exclusion criteria of controls.^20^^,^^22^^,^^23^ All studies used reliable analytical methods. Atomic absorption spectroscopy (AAS) techniques were used in 5 studies to assess mercury levels,^17^^,^^18^^,^^22^, ^23^, ^24^ while mass spectrometry-based techniques were used in 3 studies,^21^^,^^23^^,^^25^ one study used atomic emission spectroscopy^20^ and one used direct mercury analyzer.^17^ However, five studies failed to provide method performance parameters.^17^^,^^18^^,^^21^^,^^23^^,^^24^ Only two studies adjusted for major confounders in the analysis or recruitment of participants.^17^^,^^20^ One study reported on health outcomes by means of a survey but did not provide information on its validation.^21^ One study did not report on their findings thoroughly,^19^ as it failed to include the results for the control group and omitted one of the health outcomes specified in the methodology. None of the included studies reported on any potential missing data in their final analysis.

## Discussion

This systematic review provides an up-to-date synthesis of occupational mercury exposure among dental health workers and its potential health effects. We identified nine papers reporting on mercury biomonitoring data among dental health workers. Notably, all but one of the studies included in this review were conducted in LMICs, which aligns with the broader global trend of mercury use. Developed countries have moved away from mercury-based materials, opting for alternatives such as resin composites. For instance, the European Union has announced a complete ban on dental amalgam, effective in 2025.^26^ Data from New Zealand show a decrease in amalgam for direct restorations from 52.3% in 1998 to 7.1% in 2017.^27^ Similarly, the overall rate of dental amalgam restoration placements in the general population declined from 21.8% in 2017 to 4.1% in 2023 in the United States.^28^ In comparison, dental amalgam was still being used by 36.9% of dentists in Turkey,^29^ 41.6% of dentists in Pakistan,^30^ and accounted for 57.5% of dental restorations placed in Nigeria,^31^ as LMICs continue to rely on mercury-based materials. These variations in country and regional use of amalgam are driven by various factors such as local customs and norms, the general level of prosperity, the availability of dental care and dental restoration materials, the cost of dental care in general as well as the cost of alternative filling materials in particular, the influence of the insurance industry on dental costs and alternatives, and the level of public awareness of possible health and environmental effects.^3^

Overall, dental professionals consistently exhibited higher mercury levels in biological matrices compared to control groups. However, mercury levels in dental workers were lower than the recommended occupational exposure limits, suggesting no risk of occupational intoxication. Several factors contribute to the low levels observed, including the reduced use of mercury in dental procedures, as current guidelines from dental societies advocate for a limited use of dental amalgam.^32^^,^^33^ Additional factors that may explain the lower mercury levels include improved compliance with personal protective equipment protocols, the shift to encapsulated dental amalgam, which has a reduced potential for mercury release and is less prone to spillage and accidental exposure. Only one study in Egypt^18^ found higher levels of mercury in urine than the American Conference of Governmental and Industrial Hygienists’ Biological Exposure Index (BEI) of 20 μg/g creatinine.^34^ The high mercury levels among Egyptian dentists were attributed to high-risk behaviours such as mercury heating and mixing by hand in the latter study. We also note that all countries included in this review are parties of the Minamata Convention except for Egypt.^35^

The relationship between workplace factors and mercury levels could not be confirmed. Some studies identified associations between mercury levels and amalgam use frequency, workload or years of experience,^20^^,^^25^ whereas others reported no significant relationship.^19^^,^^22^^,^^23^^,^^25^ These inconsistencies may be explained by some methodological limitations. For instance, most studies did not report on adherence to personal protective equipment protocols, which likely contributes to mitigating exposure and may account for both the relatively low mercury concentrations observed and the absence of significant associations with work-related determinants of exposure. Additionally, given mercury’s relatively short biological half-life in blood and urine, measured levels primarily reflect recent rather than cumulative occupational exposure. Consequently, the absence of significant associations may result from sampling not being temporally aligned with periods of higher exposure, particularly considering the generally low concentrations observed. Overall, the inconsistent associations with work factors likely reflect methodological variability between the studies rather than the absence of an occupational effect. Current evidence does not allow identification of consistent predictors of mercury burden among dental personnel in the absence of a standardized approach to assessing exposure determinants.

Findings from environmental monitoring studies indicate that mercury vapor concentrations in dental clinics may exceed recommended safety limits. Measurements taken in Iraqi dental clinics showed concentrations of mercury vapor above ACGIH recommended threshold limit value (TLV) of 0.025 mg/m³ averaged over an 8-hour workshift.^17^^,^^34^ In contrast, air monitoring performed in dental clinics in India^21^ and Egypt^18^ was below the ACGIH threshold. Air concentrations can vary based on several factors including the frequency of amalgam manipulation and the handling of amalgam waste, which may continue to emit mercury vapor if not stored properly, while proper handling and storage of amalgam, as well as adequate ventilation system, the presence of high-volume suction, and the use of amalgam separators can ensure that the recommended threshold limits are not exceeded.^12^^,^^36^, ^37^, ^38^, ^39^, ^40^, ^41^ However, as these parameters were not documented in the included studies, it remains difficult to compare findings across settings or to identify the specific factors contributing to the observed differences in mercury vapor levels. Interestingly, opposite trends were observed in biological monitoring data. Egyptian dentists exhibited urinary mercury levels exceeding ACGIH reference values, whereas this was not the case for Iraqi dentists. This discrepancy may be explained by the fact that stationary air monitoring may underestimate actual exposure in comparison with measurements taken directly in the dentists breathing zone.^42^ Additionally, air monitoring alone does not necessarily reflect the actual dose absorbed by workers, which depends on factors such as duration of exposure and use of personal protective equipment. None of the studies conducted sampling over a full 8-hour work shift nor did they report these parameters. The presence of mercury in wastewater samples from dental clinics further supports concerns about improper disposal practices contributing to environmental contamination. According to the United States Environmental Protection Agency, dental offices are the largest source of mercury in sewage treatment plants.^43^ These findings reinforce the need to adhere to best amalgam handling practices to mitigate environmental contamination.

Various health effects were reported across the included studies. Mercury exposure was associated with changes in liver function, immune response, pregnancy outcome and oxidative stress.^17^, ^18^, ^19^^,^^24^ The potential adverse and toxic effects of dental amalgam have long been a subject of concern. However, current evidence does not support an increased risk of systemic diseases among patients with dental amalgam restorations.^44^^,^^45^ In the case of dental health workers, the reported health associations in the included studies should be interpreted cautiously due to multiple methodological limitations. Most of the included studies were cross-sectional and/or based on small sample sizes, which restricts the validity of the findings and reduces the ability to detect long-term adverse effects from chronic exposure. Furthermore, the biomarkers of effect used were often nonspecific to mercury and could be influenced by uncontrolled confounding factors. Given the low mercury concentrations observed in biological matrices, systemic effects remain unlikely.

### Limitations and recommendations for future research

The existing body of literature on occupational exposure to mercury is relatively limited and requires further exploration. For instance, there are geographical disparities in available biomonitoring data as studies were predominantly conducted in Asia. Most studies had a relatively small sample size and included both dentists and dental assistants but did not stratify mercury exposure measurements based on occupation. Dentists and dental assistants are exposed to mercury through different pathways, with dentists primarily exposed during amalgam placement and removal, while dental assistants are also exposed during preparation and sterilization processes. Additionally, there has been limited focus on the work-related factors that influence mercury exposure, which hinders the ability to conduct effective risk assessments. Similarly, when environmental monitoring was performed, no correlation with internal dose or working factors was considered.

Future research should address these gaps by employing robust methodologies, with clear justification of sampling methods, control for confounding factors, and detailed analytical procedures to improve transparency and replicability. Additionally, it is crucial to perform environmental monitoring and assess health outcomes to strengthen the body of evidence on occupational mercury exposure in dentistry and its health impacts.

## Conclusion

This systematic review highlights the persistent occupational exposure of dental professionals to mercury and its potential health implications. Despite global efforts to phase down mercury use in dentistry, biomonitoring studies indicate ongoing exposure risk, especially in LMICs. The limited number of papers included in this review, while reflective of the available research, sheds light on a significant gap in the scientific literature regarding exposure levels and highlights the need for more comprehensive data. This is of particular importance in the context of LMICs, where inconsistent regulatory enforcement, limited resources and oral public health’s low prioritization hinder phase-down efforts. Further research is needed to refine biomonitoring methodologies and assess possible long-term health effects while considering regional disparities and specificities. Until global mercury phase-down goals are fully achieved, continued surveillance of occupational exposure in dentistry remains critical to safeguarding dental professionals’ health.

## Authors' contributions

IBK: Writing – original draft, Writing – review and editing, Methodology, Formal analysis, Data curation. AM: Writing – review and editing, Methodology, Formal analysis, Data curation. RCD: Validation, Writing – review and editing. LG: Validation, Writing – review and editing. SEJ: Validation, Writing – review and editing, Supervision. YFZ: Conceptualization, Methodology, Validation, Writing – review and editing, Supervision.

## Data availability statement

All data used in this systematic review were extracted from publicly available original manuscripts. The data supporting the findings of this study can be accessed by referring to the cited publications listed in the manuscript’s reference section.

## Declaration of competing interests

The authors declare that they have no known competing financial interests or personal relationships that could have appeared to influence the work reported in this paper.

## Appendix Supplementary materials

Image, application 1

## Footnotes

Supplementary material associated with this article can be found in the online version at doi:10.1016/j.identj.2026.109560.
