# Supplementary material for: Biomonitoring of Occupational Exposure to Mercury Among Dental Health Workers in LMICs: A Systematic Review
Source: Int Dent J. 2026 May 20;76(4):109560. doi: 10.1016/j.identj.2026.109560 (PMC13213647; doi:10.1016/j.identj.2026.109560)
Supplement: Supplementary file 1 [file mmc1.docx]

**Supplementary Material 1: Search Strategy**

| PUBMED | ("dental staff"[MeSH Terms] OR "dentist*"[Title/Abstract] OR “dental assistant*"[Title/Abstract] OR "dental hygienist*"[Title/Abstract] OR "dental technician*"[Title/Abstract]) AND ("Mercury"[MeSH Terms] OR "Mercury"[Title/Abstract]) |
| --- | --- |
| SCOPUS | TITLE-ABS-KEY (dentist* OR "dental assistant*" OR "dental hygienist*" OR "dental technician*") AND TITLE-ABS-KEY ( mercury ) |
| WEB OF SCIENCE | dentist* OR "dental assistant*" OR "dental hygienist*" OR "dental technician*" (Topic) and mercury (Topic) |

**Supplementary Material 2: Summary of Risk of Bias Assessments**

|  | Risk of bias domains and ratings | | | | | | |
| --- | --- | --- | --- | --- | --- | --- | --- |
|  | Key RoB Criteria | | | Other RoB Criteria | | | |
|  | Detection bias: Were exposure and outcome measured reliably? | | Confounding Bias: Did the study design or analysis account for important confounding and modifying variables? | Selection Bias: Did selection of study participants result in appropriate comparison groups? | Attrition Bias:  Were outcome data complete without attrition or exclusion from analysis? | Reporting Bias:  Were all measured outcomes reported? | Other Bias:  Was statistical analysis appropriate? |
|  | Can we be confident in the exposure characterization? | Can we be confident in the outcome assessment? |  |  |  |  |  |
| Jamil & al.,  2016 | ++ | ++ | + | - | NR. | ++ | + |
| Al-Zubaidi & al.,  2017 | ++ | + | ++ | ++ | NR. | ++ | - |
| El-Badry & al., 2018 | ++ | + | - | ++ | NR. | ++ | + |
| Wijesekara & al.,  2018 | ++ | + | -- | -- | NR. | ++ | -- |
| Yazdanian & al.,  2020 | ++ | + | - | ++ | NR. | ++ | + |
| Tuček & al.,  2020 | ++ | ++ | -- | -- | NR. | ++ | + |
| Girgin & al.,  2022 | ++ | ++ | -- | + | NR. | - | + |
| Zebbiche & al.,  2022 | ++ | ++ | - | NA. | NR. | ++ | + |
| Mawari & al.,  2024 | ++ | - | -- | ++ | NR. | ++ | - |

| ++ Definitely Low Risk |
| --- |
| + Probably Low Risk |
| - Probably High Risk |
| -- Definitely High Risk |
| NR. Not reported |
| NA. Not applicable |
